# Simultaneous bilateral midshaft clavicle fractures (Allman Type I): Case series

**DOI:** 10.1097/MD.0000000000040638

**Published:** 2024-11-29

**Authors:** Chaode Cen, Yuehua Xie, Mao Liu, Yan Wu, Aixin Cao, Daqing He

**Affiliations:** a Department of Orthopaedics, The Beijing Jishuitan Hospital Guizhou Hospital, Guiyang, China; b Department of Orthopaedics, People's Hospital of Weining Yi, Hui and Miao Autonomous County, Bijie, China; c Department of Clinical Medicine, Jinzhou Medical University, Jinzhou, China.

**Keywords:** Allman classification, bilateral clavicle fractures, case report, open reduction and internal fixation

## Abstract

**Rationale:**

A simultaneous bilateral fracture of the middle clavicle is a very rare injury in clinical practice, and the necessity of surgical intervention and treatment modality remains a topic of debate.

**Patient concerns:**

We report a case of multiple injuries in a 40-year-old woman following a road traffic accident and another case of polytrauma in a 46-year-old man following a collision injury.

**Diagnoses:**

The radiographs of the bilateral shoulder joints and the 3-dimensional reconstruction CT of the chest showed the fractures in the middle third of the clavicle on both sides. In addition, concurrent injuries were not overlooked.

**Interventions:**

Until the patient’s life-threatening injury is prioritized and the patient’s vital signs are stable, patients were treated with the anatomical locking plate to stabilize the bilateral clavicle fractures. Progressive functional exercises were implemented following the operation.

**Outcomes:**

At the 2-month follow-up examination, the patients showed excellent range of motion and functional outcomes.

**Lessons:**

Given the rarity of this combined injury, it is crucial to minimize the duration of functional impairment and encourage early functional exercises for both shoulder joints. We recommend that surgical indications be judiciously relaxed to allow for open reduction and internal fixation using locking plates.

## 1. Introduction

The clavicle is a double-curved S-shaped bone and the only long horizontal bone that connects the axial and upper girdle bones.^[1]^ Clavicle fractures are common and account for 5% to 10% of all fractures of the human skeletal system.^[2]^ Nevertheless, bilateral clavicle fractures are rarely reported in the existing literature, with an incidence of < 0.5% of all clavicle fractures.^[3]^ Bout^[4]^ reported an overall incidence between 0.011% and 0.017%. Epidemiological studies show that 70% to 82% of all clavicle fractures occur in the middle third of the clavicle, 10% to 16% in the lateral third and only 3% in the medial third.^[5]^ The mechanism of injury in the majority of cases is axial loading by a direct insult to the shoulder girdle and in the remaining cases a fall on the outstretched hand.^[6]^ Bilateral clavicle fractures occur more frequently due to high-energy trauma or trauma with multiple injuries, which can be easily overlooked or ignored clinically and delay treatment.^[7]^ In high-energy trauma and bilateral shoulder compression injuries, proper clinical assessment and chest radiographs including both shoulder joints should be performed, and bilateral clavicle fractures should be actively sought.^[8]^

There is no general consensus on whether this injury should be treated surgically or non-surgically, and the treatment modality of bilateral clavicle fracture.^[9]^ The use of a number of different devices, including Kirschner wires, Knowles nails, Hagie nails, Rockwood nails, titanium elastic nails, screw intramedullary flexible nail and Anser clavicle nails, has been described.^[10]^ Most of these patients have a good long-term functional outcome. In this article, we report a case of a bilateral Allman Type I clavicular fracture in a 40-year-old female patient, whom we treated surgically to limit the duration of functional disability, with open reduction and internal fixation using anatomical locking plates and screws on both sides.

## 2. Case presentation

We have encountered 2 cases of bilateral clavicle fractures with different modes of trauma. One patient had a tricycle traffic accident while the another case suffered a crush injury between a wall and a frightened cow. All of the fractures united well after surgical treatment with the return of normal functional activity.

### 2.1. Case 1

A 40-year-old woman was admitted to hospital following a road traffic accident and presented with pain, swelling and bone instability in both mid-clavicular regions. In taking the full history of the injury, the patient and the tricycle fell to the ground, the right side of the upper limb was stretched during the fall, resulting in a clavicle fracture by violent conduction, after which the outside of the left shoulder hit the ground, causing a clavicle fracture. On admission, the patient was conscious and her vital signs were stable, her answers to the questions were relevant and she was co-operative during the physical examination. The chest pain worsened with breathing and coughing, and the chest compression test was positive. The patient was found to have swelling and deformation in the area of the collarbone on both sides. A soft tissue contusion can be seen on the cheek. No skin lesions or ecchymoses were noted in the bilateral clavicle region. Localized tenderness of the shoulders is evident and the sensation of bone friction can be felt. The movement of the shoulders was clearly restricted. The neurological status and vascular status of the bilateral upper extremity were normal. Other physical tests revealed no abnormalities.

**Figure 1.**
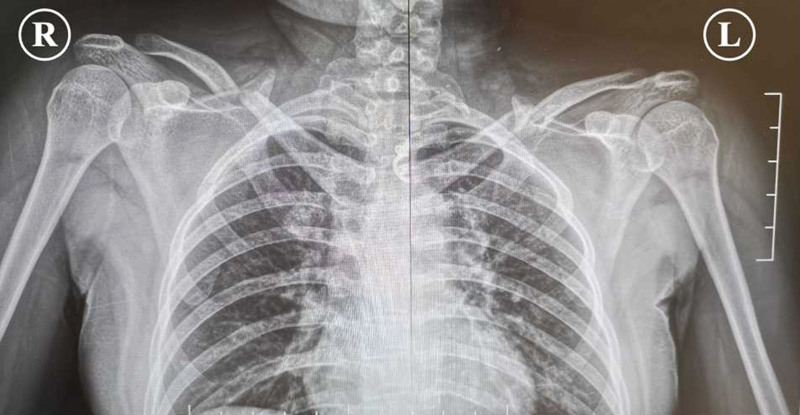
X-ray images on initial presentation: bilateral fractures of the middle clavicle.

**Figure 2.**
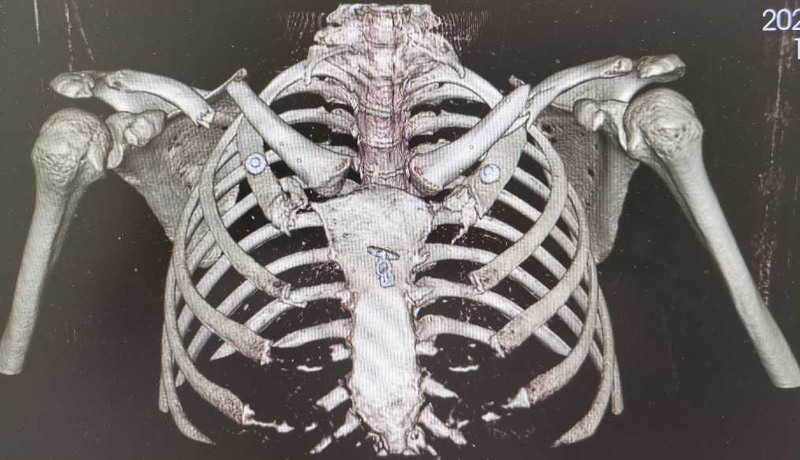
Three-dimensional CT scan on initial presentation: bilateral fractures of the middle clavicle and bilateral incomplete fractures of the 2nd and 3rd ribs. CT = computed tomography.

The patient was sent for a posteroanterior radiograph of the bilateral shoulder joints (Fig. 1) and a 3-dimensional CT scan of the chest (Fig. 2) to document the extent of the damage. The radiographs showed displaced bilateral fractures of the middle clavicle, and the CT scan surprisingly revealed bilateral fractures of the 2nd and 3rd incomplete ribs with a posterior lung contusion, without injury to the great vessels or other structures. It was decided to treat the bilateral fractures of the middle clavicle surgically, while the rib fractures were treated conservatively.

**Figure 3.**
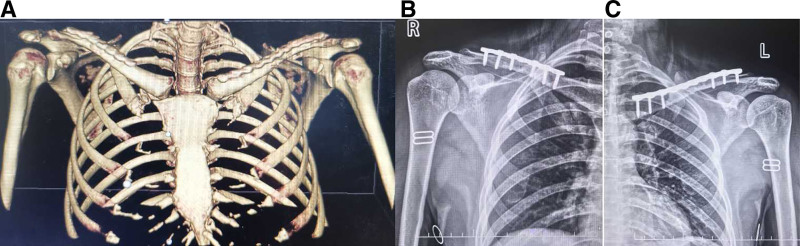
Postoperative 3-dimensional CT reconstruction of the chest after open reduction and internal fixation and X-rays of both shoulder joints 2 months after surgery. CT = computed tomography.

**Figure 4.**
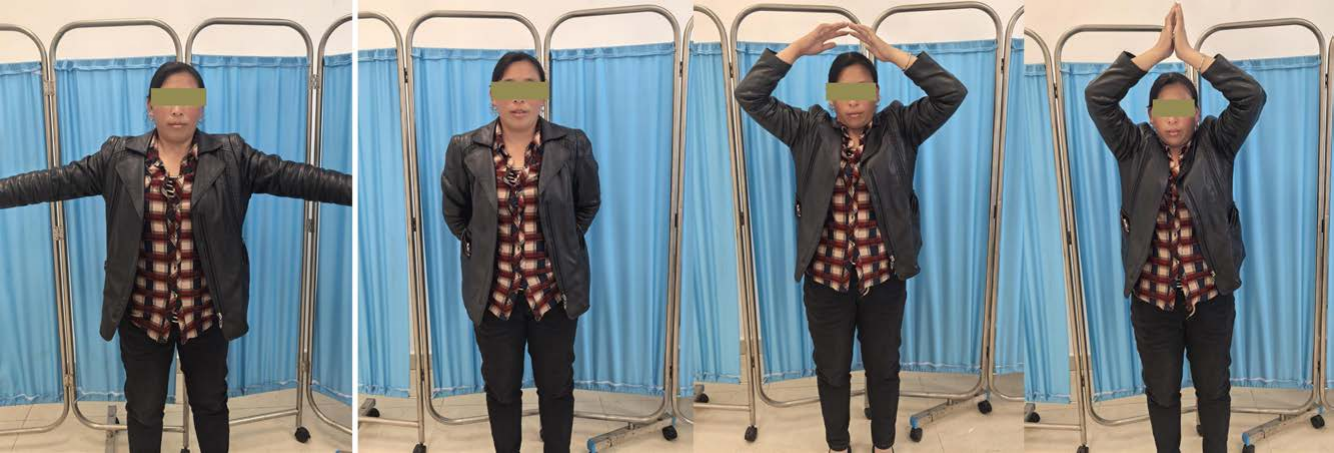
The patient’s clinical follow-up photos 8 weeks after the trauma show a good result in terms of range of motion.

The operation was performed on the third day after the injury under general anesthesia with the patient lying in the beach chair position. An incision was made over the clavicle fracture. A dissection was performed to expose the fracture site. After reduction of the fracture, the left side was fixed with a 10-hole plate and the right side with a 7-hole anatomical plate. No additional approval from our ethics committee was required, as case reports are exempt from ethical approval in our institution. Written consent was obtained from the patient for the publication of this case report and the accompanying images. The postoperative radiographs showed that the fracture reduction was satisfactory and the position and length of the internal fixation was appropriate (Fig. 3A). Two months after the operation, the X-ray of both shoulder joints showed callus growth on the bilateral clavicle fractures, and the internal fixation was reliable (Fig. 3B, C). Postoperatively, both shoulders were immobilized with a cuff and sling for 4 weeks. Patients are encouraged to actively practice flexion and extension of the elbow and wrist. Four weeks after the operation, the straps were removed and progressive active-supported physiotherapy for the shoulder joints was initiated. Two months after surgery, the shoulder is fully elevated and abducted and the patient’s daily activities have normalized (Fig. 4).

### 2.2. Case 2

**Figure 5.**
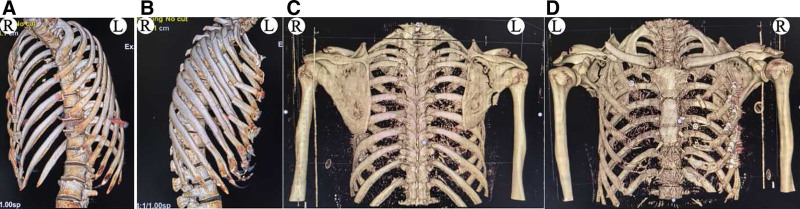
CT scan of chest showing: (A) and (B) bilateral multiple rib fractures; (C) the left scapular fracture; and (D) bilateral clavicle fractures. CT = computed tomography.

A 46-year-old man was trapped between a frightened cow and a wall while cleaning the cattle shed. He was conscious when he came to the hospital complaining of bilateral chest pain, tightness in the right chest, neck and back pain, pain and swelling in the bilateral acromioclavicular regions. Multiple skin and soft tissue contusions were noted on the chest and face. After admission, CT scan of the brain, facial skull, chest and thoracic vertebrae in the emergency department revealed bilateral multiple rib fractures and right haemopnemothorax, bilateral lung contusion and bilateral pleural effusion, bilateral clavicle and left scapula fractures (Fig. 5) as well as multiple fractures of the transverse process, the spinous process and the vertebrae in the cervical and thoracic spine.

**Figure 6.**
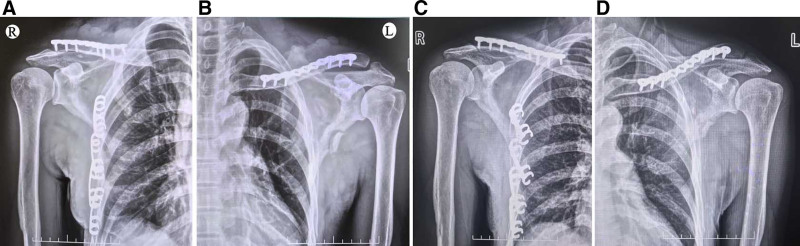
The anteroposterior X-rays of bilateral shoulder joints immediately after the operation and 2 months later.

The patient was preferably admitted to the Department of Thoracic Surgery for open reduction and internal fixation of right multiple rib fractures and closed thoracic drainage. The patient was referred to our department for open reduction and internal fixation of bilateral clavicle fractures when his chest condition was stable 8 days after surgery. Although the patient had a floating shoulder injury on the left side, no surgery was performed for a scapular fracture as there was no significant displacement of the inner and outer lateral walls of the scapula. Written consent was obtained from the patient for the publication of this case report and the accompanying images. Figure 6A, B showed satisfactory reduction and fixation of bilateral clavicle fractures after surgery and no obvious displacement of the scapular fractures. Figure 6C, D showed the callus growth and good healing in the bilateral clavicle fractures and the left scapula fracture 2 months after surgery.

**Figure 7.**
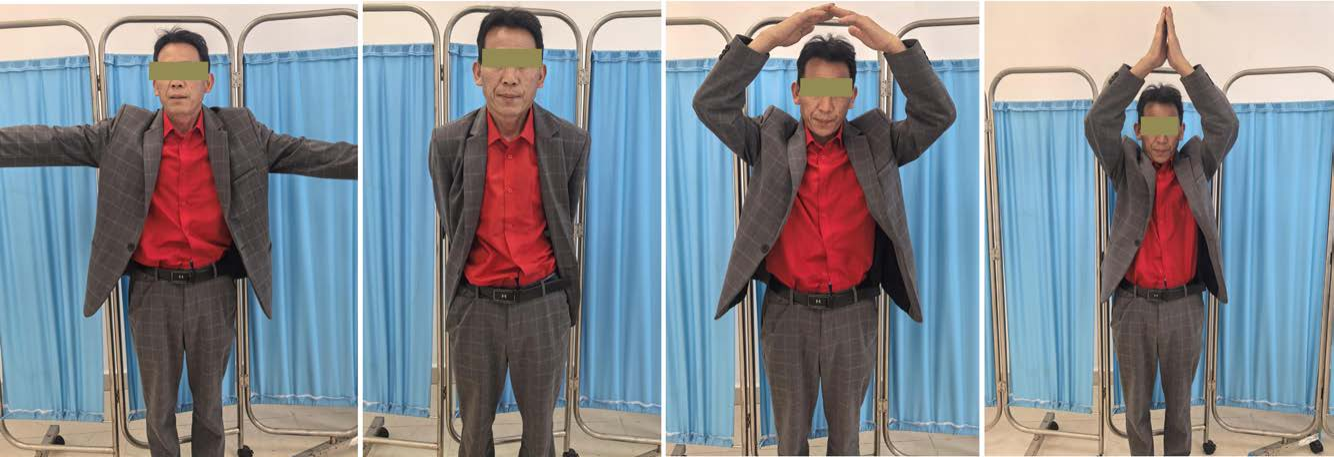
Free and full mobility of the shoulder joints was achieved 2 months after the operation, with a full constant score for the bilateral shoulder joints.

After surgery, both upper limbs were suspended with forearm straps for 8 weeks, analgesics were administered to relieve pain and both shoulder joints were actively elevated on the third day after the operation while lying down with hands crossed. The patient was discharged from our department 6 days after the operation and received a rehabilitation exercise program for follow-up treatment. Two months after surgery, the abduction and lifting functions of the patient’s shoulder joint were fully restored and the patient was able to resume his daily routine (Fig. 7).

## 3. Discussion

Clavicle fractures are common, but bilateral clavicle fractures are rare and account for only 0.43% of all clavicle fractures.^[11]^ They usually occur after high energy trauma (road traffic accidents, falls from height) and are therefore associated with other severe concomitant injuries such as severe chest injuries (multiple rib fractures, sternal fractures, pulmonary contusion, haemopneumothorax, etc), intra-abdominal injuries, brachial plexus injuries, scapular fractures, humeral fractures, spinal fractures, etc.^[12]^ The mechanism of bilateral clavicle fractures differs from that of a unilateral clavicle fracture. Bilateral traumatic clavicle fractures are often caused by a compressive force on both shoulder girdles, direct blows to both shoulder girdles or an indirect blow such as a fall on the shoulder.^[13]^ In addition to fractures caused by high-energy violence, pathological fractures caused by metabolic bone disease and long-term exposure to radiation, stress fractures or fatigue fractures, and bilateral clavicle fractures caused by electric shock can also occur.^[14–16]^

Wei et al^[17]^ described 4 possible injury mechanisms for bilateral clavicle fractures. The first is that the patient was hit by a car, the bilateral shoulder joints are loaded in a slight anteflexion and abduction position, and the external force is transmitted to the bilateral clavicle via the upper extremity and glenohumeral joints. This often leads to an Allman Type I + Type I clavicle fracture. The second is that 1 side of the upper limb is stretched during the fall, resulting in a clavicle fracture by violent conduction, whereupon the outside of the other shoulder strikes the ground and causes a clavicle fracture. This often results in an Allman Type I + Type II or Type II + Type II or Type I + Type I clavicle fracture. Third, 1 shoulder landed in the retracted position and the other shoulder was crushed by a heavy object. This often leads to an Allman Type II + Type II clavicle fracture. Fourthly, the car collided directly with the bilateral clavicle, resulting in bilateral clavicle fractures. This often leads to an Allman type III + type III clavicle fracture. The injury process in the first typical case includes the second injury mechanism and the injury process of another typical case is similar to that of the fourth injury mechanism.

In clinical practice, it is common to treat uncomplicated or non-displaced clavicle fractures conservatively. Surgical treatment is unanimously indicated for open fractures or in cases of skin injuries, neurovascular complications and a middle distal clavicle fracture with injury to the coracoclavicular ligament.^[18]^ Nevertheless, there is no general consensus on the treatment modality for bilateral clavicle fractures. A conservative approach to the treatment of bilateral clavicle fractures has been associated with more pain, short-term stiffness, nonunion and shoulder dysfunction.^[19]^ Based on the current literature, most authors recommend surgical treatment for bilateral clavicle fractures, especially in cases with impaired respiratory function, to improve respiratory function and reduce the duration of functional impairment associated with conservative treatment.^[1]^

The gold standard for fixation of the clavicle is still open reduction and internal fixation with either reconstruction plates, locking T-plates with stable angles, dynamic compression plates, intramedullary devices or a clavicular hook plate.^[20–22]^ The hook plate is suitable for elderly patients with a distal clavicular fracture in combination with a coracoclavicular ligament rupture.^[23]^ Locking plates and anchors with pre-tensioned sutures may be considered in young patients with fractures of the middle distal clavicle and tears of the acromioclavicular or coracoclavicular ligament.^[24]^ Although open reduction and intramedullary fixation of clavicle fractures is minimally invasive and simple, common disadvantages of intramedullary fixation are hardware irritation, protrusion, shortening of the clavicle (telescopic effect) and migration of the implant. In our opinion, it cannot provide sufficient stability, support satisfactory postoperative pain relief and allow early exercise of shoulder function. We have therefore opted for open reduction and fixation with locking plates for bilateral clavicle fractures.

In the case of a unilateral clavicle fracture, the patient can use the healthy limb to support the functional exercises of the shoulder joint on the affected side and at the same time carry out some daily activities. Patients with bilateral clavicle fractures have limited mobility of both shoulder joints and early active functional exercise should be done with caution as excessive and over-exercising can lead to loosening of the internal fixation and displacement of the fracture. We recommend that patients interlace their fingers to raise their shoulders on both sides when lying down. The significantly increased muscle strength and the force of gravity when standing have a negative effect on the plate and the fracture end. The passive exercise of the shoulder joint is emphasized within 4 weeks, then both upper limbs were suspended with a sling in the exercise interval. Passive exercise was gradually replaced by active exercise after 4 weeks. Progressive functional exercises can improve the range of motion of bilateral shoulder joints and prevent the occurrence of shoulder stiffness.

It is essential to acknowledge the inherent limitations of this case series. Firstly, the duration of our follow-up was limited, and we did not perform a long-term assessment of shoulder function. Secondly, simultaneous bilateral midshaft clavicle fractures are frequently associated with other injuries, which may impact treatment outcomes and prognosis assessment. Finally, each case exhibits unique characteristics, including patient age, sex, health status, and fracture type, this heterogeneity may complicate the replication of our findings in other similar cases.

## 4. Conclusion

Bilateral clavicle fractures are exceptionally rare and are typically associated with polytrauma. The mechanisms of injury for bilateral clavicle fractures differ from those of unilateral fractures. We recommend that the surgical indications be judiciously relaxed to permit open reduction and internal fixation with a locking plate, thereby reducing the duration of functional disability, enhancing respiratory function, and improving clinical outcomes. Following a structured rehabilitation program, patients can achieve restoration of shoulder joint function to pre-injury levels.

## Acknowledgments

This study was supported by the Guizhou Provincial Science and Technology Foundation, Science and Technology Plan Project (Qian Ke He Ji Chu-ZK[2021] General 389) and the Guizhou Provincial Basic Research Program (Natural Science; Qian Ke He Ji Chu-ZK[2024] General 579.

## Author contributions

**Conceptualization:** Daqing He.

**Funding acquisition:** Chaode Cen.

**Investigation:** Mao Liu, Yan Wu, Aixin Cao.

**Methodology:** Daqing He, Yuehua Xie.

**Project administration:** Chaode Cen.

**Supervision:** Daqing He.

**Writing – original draft:** Chaode Cen, Yuehua Xie.
